# Galactosemia: Biochemistry, Molecular Genetics, Newborn Screening, and Treatment

**DOI:** 10.3390/biom12070968

**Published:** 2022-07-11

**Authors:** Mariangela Succoio, Rosa Sacchettini, Alessandro Rossi, Giancarlo Parenti, Margherita Ruoppolo

**Affiliations:** 1 CEINGE-Biotecnologie Avanzate s.c.ar.l., 80145 Naples, Italy; mariangela.succoio@unina.it (M.S.); sacchettini@ceinge.unina.it (R.S.); 2 Department of Translational Medical Sciences, University of Naples Federico II, 80131 Naples, Italy; alessandro.rossi@unina.it (A.R.); parenti@unina.it (G.P.); 3 Telethon Institute of Genetics and Medicine (TIGEM), 80078 Pozzuoli, Italy; 4 Department of Molecular Medicine and Medical Biotechnology, University of Naples Federico II, 80131 Naples, Italy

**Keywords:** galactosemia, newborn screening, galactose metabolism, classical galactosemia, gene-based therapies, small molecules therapies

## Abstract

Galactosemia is an inborn disorder of carbohydrate metabolism characterized by the inability to metabolize galactose, a sugar contained in milk (the main source of nourishment for infants), and convert it into glucose, the sugar used by the body as the primary source of energy. Galactosemia is an autosomal recessive genetic disease that can be diagnosed at birth, even in the absence of symptoms, with newborn screening by assessing the level of galactose and the GALT enzyme activity, as GALT defect constitutes the most frequent cause of galactosemia. Currently, galactosemia cannot be cured, but only treated by means of a diet with a reduced content of galactose and lactose. Although the diet is able to reverse the neonatal clinical picture, it does not prevent the development of long-term complications. This review provides an overview of galactose metabolism, molecular genetics, newborn screening and therapy of galactosemia. Novel treatments for galactosemia currently being investigated in (pre)clinical studies and potentially able to prevent long-term complications are also presented.

## 1. Galactose Metabolism

Galactosemia is a rare inborn metabolic disorder of carbohydrate metabolism presenting with multi-organ involvement and is potentially fatal if not diagnosed on time. Biochemically, it is characterized by hypergalactosemia, a condition associated with an increased blood level of galactose. Galactose is a natural aldohexose sugar that is found in all living organisms both as endogenous galactose and as galactose derived from the diet, mainly through (human) milk and dairy products, in the form of lactose, a disaccharide formed by glucose and galactose. Not only is galactose a source of energy but also plays a structural role, as it is a constituent of various macromolecules (e.g., glycolipids and glycoproteins) [1]. In physiological conditions, after (ga)lactose ingestion, it is absorbed across the small intestine by enterocytes and is actively transported across the brush border membrane by the sodium–glucose cotransporter SGLT1. It enters the liver via the portal vein and is internalized by hepatocytes through the glucose transporter 2 (GLUT2) transmembrane carrier protein. Eventually, it is converted to glucose-1-phosphate (Glc-1-P) via the Leloir pathway to either produce energy for several tissues of the body [2,3] or allow the biosynthesis of complex molecules.

**Figure 1:**
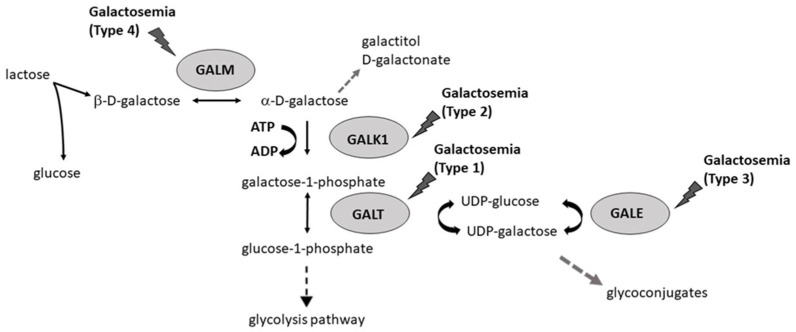
Schematic representation of the Leloir pathway and associated reactions. Several forms of galactosemia can be caused by deficiency of GALM, GALT, GALK1, or GALE.

Galactosemia occurs due to disruptions in various steps of galactose metabolism, including deficiencies in the enzymes in the Leloir pathway, portosystemic shunts, cholestasis, and other metabolic disorders such as Fanconi–Bickel syndrome [4]. Four main enzymes catalyze a specific reaction in the Leloir pathway (Figure 1): galactose mutarotase (GALM, EC 5.1.3.3), galactokinase (GALK1, EC 2.7.1.6), galactose-1-phosphate uridylyltransferase (GALT, EC 2.7.7.12), and UDP-galactose 4′-epimerase (GALE, EC 5.1.3.2) Thus, galactosemia, can be classified into four different forms (1–4) according to the altered galactose metabolic enzyme: (i) type 1 galactosemia; (ii) type 2 galactosemia; (iii) type 3 galactosemia; (iv) type 4 galactosemia [5].

The first enzyme of the galactose metabolic pathway is GALM an aldose epimerase that catalyzes the reversible interconversion between β- and α-D-galactose. The latter is then converted to galactose-1-phosphate (Gal-1-P) by GALK. GALK is a protein belonging to the superfamily of small molecule kinase enzymes structurally similar named GHMP (galactokinase, homoserine kinase, mevalonate kinase, and phosphomevalonate kinase) [6], which phosphorylate substrates at the expense of ATP. GALT is involved in the subsequent metabolic step. GALT is a homodimer belonging to the histidine triad (HIT) superfamily, in which each monomer consists of 379 amino acids and has an active site containing the His-Pro-His functional motif at the interface between the two subunits [7]. GALT converts Gal-1-P into glucose-1-phosphate (Glc-1-P) with the formation of UDP-galactose (UDP-Gal) from UDP-glucose (UDP-Glc) by a “ping-pong” mechanism.

UDP-galactose 4-epimerase (GALE), the fourth enzyme of the Leloir pathway, belongs to the short-chain dehydrogenase/reductase (SDR) superfamily and catalyzes the reversible conversion of UDP-Gal into UDP-Glc with NAD+ as a cofactor. UDP-Glc is essential for the enzymatic activity of GALT. GALE is also responsible for the interconversion of UDP-N-acetylglucosamine (UDP-GlcNAc) and UDP-N-acetylgalactosamine (UDP-GalNAc), required for the galactosylation of complex molecules and the synthesis of different glycoproteins/glycolipids [5].

In case the Leloir pathway is impaired, galactose accumulates in cells, and alternative galactose metabolism pathways are activated. These pathways result in the accumulation of toxic metabolites such as galactitol and D-galactonate, which build up in different tissues and can cause tissue damage [8].

Galactitol is a glucose epimer produced from aldose reductase, the first enzyme of the polyol pathway [9]. Physiologically, this pathway converts excess glucose into fructose through the sequential activity of two enzymes: aldose reductase and sorbitol dehydrogenase. When galactose is in excess, it enters the polyol pathway and is reduced to galactitol. Galactitol cannot be further metabolized by sorbitol dehydrogenase and poorly diffuses through cell membranes. Thus, it accumulates in cells. The intracellular increase in galactitol induces hyperosmotic and oxidative stress and is responsible for the onset of cataracts in patients with GALT and GALK deficiency [10]. Excess intracellular galactose can also be oxidized to D-galactonate. Galactose dehydrogenase converts galactose to galactonolactone, which is then converted spontaneously or enzymatically to D-galactonate [11]. D-galactonate, can be either excreted directly in the urine or enter the pentose phosphate pathway where it is converted to β-keto-D-galactonic acid and subsequently D-xylose [12].

Finally, in the presence of GALT deficiency, accumulated galactose-1-phosphate can also activate the pyrophosphorylase pathway (in particular UDP-glucose pyrophosphorylase) resulting in the UDP-Glc and uridine diphosphate (UDP)-hexose deficiency. Taken together, the activation of the above-mentioned alternative pathways contributes to increased oxidative stress and endoplasmic reticulum stress, impaired glycosylation, and altered signaling pathways [13].

## 2. Molecular Genetics of Galactosemia

Galactosemia is an autosomal recessive inherited metabolic disorder of galactose metabolism. Table 1 shows the molecular characteristics of the four different forms of galactosemia.

Type 1 galactosemia is due to mutations of the *GALT* gene mapped on chromosome 9p13 (Table 1) and is the most frequent galactosemia form with a prevalence of about 1: 60,000 live births in the general population, and 1:47,000 in the Caucasian population [19]. Variations in the *GALT* gene can lead to the absence or reduction in the activity of the GALT enzyme. Based on the residual GALT activity, three sub-variants are recognized: (1) classical galactosemia (CG), (2) clinical variant, and (3) biochemical variant also called the “Duarte variant”. CG is characterized by a GALT activity of less than 1% and is associated with severe phenotype with the development of symptoms in the neonatal period. Clinical variant of galactosemia is associated with a low residual GALT activity (usually less than 10–15%) and shows less severe neonatal symptoms and a lower risk of long-term absence or moderate complications if an appropriate diet regimen is started early. Biochemical variant is characterized by a residual GALT activity of less than 25% and the subjects are clinically asymptomatic [1,14].

More than 300 gene variants have been identified for type 1 galactosemia. c.563A>G (p.Gln188Arg) is the most frequent missense variant associated with classic galactosemia and it accounts for about 70% of all pathogenic variants identified in patients with CG in the Caucasian population [15,16]. Homozygous c.563A>G (p.Gln188Arg) variants seriously compromise GALT activity. Compound heterozygous carrying one c.563A>G (p.Gln188Arg) variant presents less severe clinical manifestations than homozygous individuals. One exception is compound heterozygosity with the missense variant p. Arg33Pro. Viggiano and colleagues demonstrated by molecular modeling programs that the heterozygous genotype p.Gln188Arg/Arg33Pro exhibits a negative effect both on the active site and on important intermolecular and intramolecular interactions [7]. Another serious and frequent variant in the Caucasian population is p.Lys285Asn with a prevalence of 54% among the allelic variants associated with CG. p.Lys285Asn is prevalent in southern Germany, Austria, and Croatia and is associated with poor prognosis for neurological and cognitive functions in both homozygosity and as compound heterozygosity with the p.Gln188Arg variant [15].

Duarte galactosemia constitutes the prevalent form of the biochemical variant of galactosemia and is approximately ten times more frequent than the classic form (about 1: 4000 live births in the Caucasian population) [17]. The variant p.Asn314Asp (c.940A>G) is associated with both Duarte allelic variants (D1 and D2). In D1, also named “Los Angeles”, the missense variant results in cis with p.Leu218Leu (c.652C>T), a synonymous mutation; this causes overexpression of the enzyme and then an increase in GALT activity (>140% vs normal); in D2, on the other hand, 940A>G results in cis with three intronic variants (c.378-27G>C, c.507+62G>A, and c.508-24G>A), and with a deletion of four pairs of bases in the untranslated region (UTR region), c.-119-116 of the GTCA [15]. In vitro studies on the molecular characterization of the Duarte D2 variant showed that 5’UTR-del induces a 55% decrease in the expression levels of the mRNA of the *GALT* gene [18].

Although CG occurs in many countries of the world, some ethnic-genotypic associations exist. p.Ser135Leu (c.404C>T) is the most common variant among African Americans and in South Africa [14], whereas two complex deletions of 5 and 5.5 kb in the *GALT* gene have been strongly linked to the Ashkenazi Jewish population [19,20].

As previously mentioned, type 2 galactosemia is due to a deficiency of the GALK1 enzyme with a frequency of 1: 1000,000 in the Caucasian population. Two galactokinase-like genes are reported in the literature [27]: *GALK1*, located on chromosome 17q25 (Table 1), which encodes galactokinase involved in galactose metabolism, and *GALK2*, located on chromosome 15q21, which encodes N-acetylgalactosamine kinase (EC 2.7.1.157) that acts as a galactokinase when galactose is present at high concentrations and may be involved in a salvage pathway for the reutilization of free GalNAc derived from the degradation of complex carbohydrates. Variants in the *GALK1* gene are associated with type 2 galactosemia and, at present, the variant spectrum comprises more than 30 variants. The most frequent disease-causing variants are: p.Pro28Thr (c.82C>A), common in gypsy individuals across Europe [21], and the Osaka variant p.Ala198Val (c.593C>T), common in the Japanese and Korean populations [22]. The main clinical manifestation of GALK deficiency is cataracts due to the accumulation of galactitol responsible for osmotic lesions in the lens fibers [28].

Type 3 Galactosemia is a very rare autosomal recessive disease caused by mutations in the *GALE* gene located on chromosome 1p36 (Table 1) encoding an enzyme belonging to the short-chain dehydrogenase/reductase (SDR) superfamily. GALE deficiency is generally classified into three different forms: generalized, intermediate, and peripheral [29]. Patients with the generalized form represent the most severe form in which there is a profound GALE deficiency in all tissues. In contrast, infants with the peripheral or intermediate form generally are asymptomatic even on a regular milk diet and are usually identified only by biochemical tests, often by newborn screening (NBS) programs [30]. In fact, in the peripheral form, GALE activity is deficient in red blood cells and circulating white blood cells, but normal or near normal in all other tissues; in the intermediate form, GALE activity is deficient in red blood cells and circulating white blood cells and less than 50% of normal levels in other tissue. Missense variants associated with the peripheral and generalized forms of GALE deficiency include: p.Lys257Arg and p.Gly319Glu in the peripheral form common in African Americans and p.Val94Met found in the generalized GALE deficiency [23,24].

Recently, type 4 galactosemia was described, which was identified through NBS in Japan [25]. Variants of the *GALM* gene (Table 1) were found in a subgroup of newborns. The *GALM* gene encodes for an aldose 1-epimerase found directly upstream of GALK1. Five variants have been identified: three nonsense and frameshift variants (p.Arg82*, p.Ile99Leufs*46, and p.Trp311*), leading to premature termination codons and the two missense variants (p.Gly142Arg and p.Arg267Gly). Based on the results of in vitro GALM expression and protein stability assays, all five variant proteins were unstable as compared to wild-type proteins [25,26].

Havva Yazici et al. [31] point out in a recent case report that the pathophysiology, clinical manifestations, and treatment modalities of type 4 galactosemia are currently unknown. In support of that, they describe a clinical case of two siblings who share the same homozygous mutation of the *GALM* gene and similar to the cases reported by Wada et al. [32], but only one of them developed symptoms (i.e., cataracts), abnormal galactose metabolites, and impaired liver function (i.e., AST and ALT). Further studies are therefore necessary to clarify whether the GALM enzyme plays an exclusive role in the Leloir pathway or is also involved in the metabolism of other monosaccharides, thus possibly explaining the phenotype heterogeneity observed in patients with GALM deficiency.

## 3. Newborn Screening for Galactosemia

Newborn screening (NBS) is an important health preventive intervention carried out on the entire population of newborns. It is performed by minimally invasive methods on dried blood spot (DBS) samples collected from the heel within the first 72 h of life [33].

The aim of NBS is to pre-emptively identify various disorders and establish a targeted management plan before the onset of symptoms. Timely treatment improves the prognosis and reduces permanent damage to the newborn and can even avoid death.

NBS was initiated in Europe in the 1960s for phenylketonuria (PKU), the most common congenital metabolic disease characterized by severe mental retardation, using the Guthrie bacterial inhibition test. In the same decade, it became possible to screen galactosemia as first described by Beutler and Beluda [33,34]. With the technological evolution, several analytical refinements were introduced such as radioimmunoassays, immunochemical assays, and more recently tandem mass spectrometry (MS/MS). By means of these methods, additional inherited disorders can be detected by NBS. However, the number of disorders included in NBS programs still largely varies worldwide [34]. For example, in Italy, NBS for galactosemia is mandatory according to national law (Law 167/2016; DM 13 October 2016; DPCM 12-1-2017).

Loeber and colleagues [34] have recently described the development and current state of NBS in Europe highlighting that there are significant differences not just in conditions screened but also in the process of confirmatory diagnostics, treatment, and follow-up of screening results. According to the authors, NBS for PKU has become almost universal. However, some diseases such as galactosemia are only included in a limited number of European NBS programs. This may be due to the (combination of) following reasons: (i) the disease can be diagnosed clinically, (ii) there is a high rate of false positives, and finally (iii) long-term complications are common despite early diagnosis [35]. From a technical point of view, NBS for galactosemia [36] can be performed by measuring total blood galactose (TGAL) or by GALT activity assay, both on a DBS using a fluorometric assay. Many screening centers use only the TGAL test as the first test of screening; other centers use both tests as first screening tests. The GALT activity assay can be used also as a second-tier test. The dosage of total galactose in DBS represents the sum of galactose and galactose 1-P, the latter is the first metabolite of the Leloir pathway; the test is based on a fluorescent method of galactose oxidase in which a series of enzymatic reactions lead to the formation of a fluorescent product directly proportional to the total galactose contained in the blood sample. The GALT activity assay determines the enzymatic activity of GALT, the most commonly affected enzyme in galactosemia by semiquantitative spectrophotometric detection of NADH or NADPH. The latter is produced through serial enzymatic reactions catalyzed by phosphoglucomutase-1 (PGM1), glucose-6-phosphate dehydrogenase (G6PD), and 6-phosphogluconate dehydrogenase (6PGD) in which NADP+ is reduced to NADPH. Consequently, the GALT activity assay is influenced by the activity of other enzymes. Therefore, considering there are several enzymes in this pathway that can cause disorders when deficiencies are present, newborn screening using the GALT activity assay may lead to misdiagnosis and be responsible for false-positive results [37].

False-positive neonatal screening for galactosemia may also be due to cases of congenital portosystemic shunts (CPSS), rare vascular anomalies resulting in communications between the portal venous system and the systemic venous circulation [38]. In fact, hypergalactosemia may occur in 13–30% of CPSS cases and is thought to derive from ingested milk and the absence of a first hepatic passage, despite the normal activity of the GALT enzyme [23,38].

False-positive galactosemia screening results can also be found in the case of glycogen storage disease type XI [36].

On the other hand, false-negative results can be obtained in case samples are not stored/collected correctly (i.e., enzyme activity assay can be subject to alteration following exposure to high temperatures and humidity) [37,38,39,40,41,42,43]. This observation is not restricted to galactosemia screening but concerns all the screening based on the evaluation of the enzymatic activity as the first test, i.e., biotinidase deficiency.

As for other metabolic diseases, false-negative galactosemia screening results can be obtained in infants that receive total parenteral nutrition or also in transfusion-treated infants who perform screening tests before seven days post-transfusion [23].

To minimize the false-positive or false-negative results in NBS for metabolic diseases, screening programs distinguish some categories of newborns that comply with specific protocols.

Many differences between the screening programs of different countries exist in the definition of special protocols. Our experience concerns the Campania Region, which reflects the Italian organization. In our system, special protocols for newborns are classified as the following: (i) low birth weight infants (less than 1.8 kg), in this case, additional DBSs are analyzed, at 14 days and 30 days after birth; (ii) blood transfusion, for which a first sample is taken prior to the start of the transfusion, and a second sample is collected one week after the suspension of the treatment; (iii) parenteral nutrition, for which a DBS is collected 48 h after treatment discontinuation [39].

Following a positive newborn screening test, the newborn is referred to a specialized clinical center that evaluates the patient’s clinical features. Clinical evaluation is followed by biochemical and molecular diagnostic confirmation.

Among the additional laboratory investigations for diagnostic confirmation of galactosemia, there are: indirect and/or direct serum bilirubin, serum ALT and AST, clotting tests, urinary reducing substances, and dosage of urinary galactitol [44].

Urine-reducing substances can point to galactosemia, but they can also be positive in other conditions such as prematurity, renal tubular acidosis, drug assumption (i.e., acid, amino acids), and other metabolic diseases. Yang and colleagues suggested that the serum or urinary dosage of galactitol by the boronic acid-based method may distinguish galactosemic patients from healthy individuals and be an important parameter for the assessment of steady-state galactose metabolism in galactosemia [45].

While the benefits of a newborn screening for galactosemia on short-term complications are known long-term complications (e.g., intellectual disability, fertility issue, bone disease) still affect patients’ outcomes and impact their quality of life [42]. The most likely cause of long-term complications is believed to be the persistent increases in metabolites due to endogenous galactose production. In addition, some authors have suggested that prenatal galactose intoxication due to maternal nutrition may lead to neonatal consequences [19].

Another debated topic is the Duarte variants of galactosemia that represent 11–50% of galactosemia cases detected by neonatal screening programs [36]. These patients have unaffected or slightly reduced GALT activity and are asymptomatic. In view that there are no reported cases of Duarte galactosemia, which have been clinically detected, some authors consider this condition benign, not requiring any treatment, while others believe that a galactose-free diet is necessary for the first year of life and has to be reintroduced only after assessing the levels of Gal-1-P in the blood [42]. In the literature, some studies report some differences in socio-emotional development, delayed memory, auditory processing speed, and liver and kidney complications among children with Duarte’s galactosemia and their unaffected siblings. To date, it is unclear whether early galactose exposure is the determining factor, or whether the restriction of galactose in the first days of life protects against the serious complications of Duarte galactosemia [46]. In addition, it should be noted that most cases of compound heterozygotes (D2/G) may have a normal NBS result. The dilemma of Duarte galactosemia has led to suggestions of lowering the cut-offs for NBS tests. Based on our experience of the population of Campania (44,334 newborns screened) and with the data currently available to us, in 2021 using a cut-off of TGAL of 7 mg/dl, we identified Duarte variants which account for about 50% of increased galactose cases (unpublished data), confirming data from the literature mentioned above [36].

## 4. Treatment

Diet is the cornerstone of the treatment of CG, GALK1 deficiency, and generalized GALE deficiency aiming at minimizing galactose intake [14,29]. Individuals with intermediate GALE deficiency should receive a galactose-restricted diet at least in infancy [29]. With only 10 patients with GALM deficiency having been reported, the need for galactose restricted diet is currently assessed on a case-by-case basis [31,32]. A galactose-restricted diet (e.g., discontinuation of breast milk or whey-based infant formula) is effective in preventing or resolving acute neonatal life-threatening symptoms (e.g., vomiting, poor feeding, lethargy, metabolic acidosis, jaundice, abnormal clotting, liver failure) [47]. A more favorable outcome has been observed in patients starting a galactose-restricted diet in the first week of life [16]. Soy-based formulas or elemental formulas are employed as the only feeding source before weaning [48]. Although casein protein hydrolysate formula contains traces of lactose, it is also considered safe in CG [47]. Recommendations on (the introduction of) complimentary food have changed over time. In the 1990s, the evidence of the presence of free galactose in a wide variety of foods resulted in highly restrictive indications [49]. More recently, a less restricted diet has not been associated with an increased risk of neurological complications [16]. Currently, animal milk and dairy products are restricted [50], while there is no evidence that the consumption of food containing trace amounts of galactose (e.g., fruit, vegetables, and legumes) has any adverse effects on patients’ outcomes [48]. Mature cheeses and soy-based products are allowed based on their relatively low galactose content (<25 mg/100 g) as compared to endogenous galactose production (>24.8 mg/kg/day in newborns, 8.4 mg/kg/day in adults) [51,52]. Conversely, there is still limited evidence to support specific age-related recommendations for the amount of galactose allowed in the diet [47]. As dietary restriction may result in calcium and vitamin D deficiency, both should be supplemented as necessary.

Since children with Duarte galactosemia do not present with clinical symptoms but are detected via NBS, there is an ongoing debate about whether dietary treatment is needed. The most common practice is not to treat individuals with Duarte galactosemia [47]. Yet, some metabolic centers prescribe a galactose-restricted diet in the first year of life [53] usually followed by a galactose challenge around age one year [17].

Irrespective of the dietary treatment approach, patients with CG are still at risk of developing long-term complications, such as intellectual disability, primary ovarian failure, and reduced bone mineral density [16]. This suggests that the development of long-term complications may depend on the combination of individual exogenous galactose tolerance (as reflected by the genotype) and other factors, such as impaired protein glycosylation, epigenetic regulation, and activation of inflammatory pathways [54]. In order to develop novel treatments able to prevent long-term complications, various options are currently being investigated in preclinical and/or clinical studies (see Table 2 for ongoing clinical trials). Therapeutic compounds can be classified into two main groups: (1) gene-based therapies and (2) small molecules.

### 4.1. Gene-Based Therapies

Gene-based therapies include gene therapy and mRNA therapy and aim at restoring GALT activity up to 10–15%, thus preventing clinical disease [14]. *GALT* gene therapy with various adeno-associated virus (AAV) vectors resulted in increased GALT levels in the liver (64–595%) and brain (3–42%) in GALT null rat pups without significant adverse effects. A reduction in galactose, galactitol, and galactose 1-P concentrations in the liver, blood, and brain as well as an improvement in cataracts were also observed [55]. Biochemical and clinical improvement was observed up to 2 months in treated rats, suggesting efficacy of gene replacement through early adulthood [56]. Restored GALT activity and protein level as well as reduced oxidative stress were also observed in fibroblasts of CG patients transduced with AAV2-CMG-hGALT [57]. Despite its potential benefit, the observed difference between liver and brain GALT activity as well as the immune response to AAV vector and genomic instability warrant further studies before gene therapy can translate into clinical practice [58].

Like other inborn metabolic disorders, the effect of mRNA therapy has also been investigated in CG. mRNA can be encapsulated in various vehicles (e.g., liposomes, nanoparticles, viruses) and delivered to the site of action where it is translated into a functional protein [59]. A single GALT mRNA dose soon after birth reduced mortality in GALT-deficient mice fed a milk diet. Additionally, a dose-dependent increase in GALT expression and activity in the liver as well as decreased galactose 1-P levels in the liver, red blood cells, and peripheral tissues were observed in GALT-deficient mice treated with GALT mRNA using lipid nanoparticles (LNP) [60]. Consistently, administration of LNP-packaged hGALT mRNA was safe and restored GALT protein levels and activity in a zebrafish model of CG [61]. Tailoring LNP to target extrahepatic tissues (e.g., brain, gonads), defining the most effective dosing interval, and assessing the mRNA-induced immune response remain important challenges to developing mRNA therapy as a treatment for CG.

### 4.2. Small Molecules

Small molecules under investigation for the treatment of CG include: (1) pharmacological chaperones, (2) enzyme inhibitors, and (3) endoplasmic reticulum (ER) stress-reducing agents.

Pharmacological chaperones are low molecular weight compounds that bind specifically to their target (protein) and stabilize it [62]. As such, they can facilitate protein folding and intracellular trafficking and/or prevent premature degradation [63]. Although pharmacological chaperones present several advantages (e.g., oral availability, potential to cross the blood–brain barrier), they cannot be used in all patients. While being effective in rescuing missense variants beyond the active site, they are not useful in case of deletion, stop gain variants, splicing variants, or active site variants [63]. Thanks to its anti-aggregation property, arginine has been studied as a potential treatment option for CG. Although initial studies suggested a potential benefit of arginine on specific GALT variants [64], subsequent research failed to confirm an effect of arginine on GALT stability [65]. As at least two binding sites (i.e., active site and binding site for allosteric modulators) in the GALT exist [64], a combination of in silico screening of predicted ligands, screening of chemical libraries, and rational screening of substrate is expected to identify novel drug candidates in the future. Yet, definition of the safety profile, appropriate (age-dependent) dosing, and specific indications (i.e., GALT variants) remain major challenges [5].

Enzyme inhibitors include galactokinase 1 (GALK1) inhibitors and aldose reductase (AR) inhibitors.

GALK1 inhibitors aim at reducing galactose 1-P accumulation, which plays a key role in the pathogenesis of CG [13]. Several candidate compounds have been identified by high-throughput screening. Among these, phenylsulfunamides have been shown to lower galactose 1-P levels in fibroblasts from CG patients [66]. More recently, non-competitive GALK1 inhibitors have been identified with the potential for further development in clinical trials [67]. As the in vivo effect of such inhibitors remains to be elucidated, additional research beyond cellular models is warranted.

AR inhibitors target galactose conversion to galactitol, which accumulates in cells leading to cell swelling and apoptosis in CG [68]. Particularly, accumulation of galactitol within the lens causes galactosemic cataracts [69]. Galactitol accumulation may also play a role in the development of neurological symptoms [68]. Originally developed for the treatment of diabetes [70], AR inhibitors decreased galactitol levels in plasma, liver, and brain and prevented cataract formation in GALT-null rat models [68,71]. Attenuation of galactitol-induced Schwann cell injury has also been observed in galactosemic rats treated with an AR inhibitor [72]. Whether AR inhibitors are able to address extraocular symptoms (e.g., cognitive symptoms, subfertility) remains to be established. Moreover, the potential effect of blocking the galactose conversion to galactitol (e.g., galactonate increase) remains to be elucidated [73].

ER stress has been demonstrated in fibroblasts from both GALT-null mice [74] and galactosemic patients [75]. Particularly, downregulation of the PI3K/Akt pathway may play a role in subfertility and ataxia [74]. Salubrinal is a eukaryotic initiation factor 2α (eIF2α) inhibitor that upregulates the cellular stress response, thus alleviating ER stress [76]. Reduced loss of both Purkinje cells in the cerebella and primordial ovarian follicles without detectable adverse effects was observed in GALT-null mice treated with Salubrinal [77]. Thus, ER constitutes a promising novel therapeutic target to be explored in CG.

### 4.3. Other Therapies

In addition to therapies (aiming at) targeting the enzyme defect, approaches addressing the clinical consequences of CG have also been developed. Indeed, despite a galactose-restricted diet, patients are still at risk of developing long-term complications.

Intellectual disability, speech delay, movement disorders, and emotional disturbance are common in CG [16]. Besides current approaches used to treat patients who develop such complications [47], transcranial alternating current stimulation (tACS), and Babble Boot Camp (BBC) are being investigated in CG [68], tACS is a form of non-invasive brain stimulation inducing long-term synaptic plasticity. This approach has proven effective in Parkinson’s disease [78] and dyslexia [79]. BBC is a preventive speech intervention program constating active parental involvement guided by a speech specialist. A trend between speech outcomes has been observed in four children with CG receiving BBC as compared to one child with CG who did not receive BBC, supporting the potential benefit of this approach [80].

Hypogonadotropic hypogonadism and primary ovarian insufficiency are common features in female patients with CG [47]. Although pregnancies in women with CG can occur [81], the chances of pregnancy are reduced [47]. Currently, two treatment options to address subfertility can be offered to patients with CG: cryopreservation and oocyte donation. Fertility preservation is only likely to be successful in very young prepubertal patients. Three fertility preservation procedures are available: ovarian tissue, mature oocyte, and/or embryo cryopreservation [82]. Even though cryopreservation reduces the ovarian reserve, this technique is now associated with an increasing success rate and low complication rate [83]. Intrafamilial oocyte donation is another approach to address subfertility in CG. Yet, several topics need to be discussed when considering this option, including the patient’s cognitive level, family relations, and medical impact [84]. As fertility preservation techniques often raise ethical questions, they should only be offered after appropriate institutional research ethics approval [82].

## 5. Conclusions

Galactosemia is a rare hereditary disease of galactose metabolism that affects the organism’s ability to convert this sugar, mainly contained in milk, into glucose. There are four types of galactosemia, depending on which enzyme of the Leloir pathway is affected. The most common and severe type is called classic galactosemia characterized by a (very) low GALT activity.

NBS for galactosemia is performed by assessing total galactose and/or GALT activity using a small amount of blood collected from the baby’s heel within 72 h of life. NBS allows the presymptomatic diagnosis with timely treatment starting. Although, more and more countries have introduced galactosemia into their NBS programs, it has still not spread universally throughout Europe, mainly due to the high rate of false-positive results.

When galactosemia is suspected, a standard galactose-free diet should be promptly started. Although dietary treatment can prevent or reduce the risk of acute clinical manifestations, it may not prevent long-term complications. In some cases, children still develop speech delays, intellectual disability, and female infertility. Several novel therapeutic approaches are being investigated to provide better treatment for galactosemia. Gene-based approaches and enzyme inhibitors appear promising. Additional studies are needed to establish a more efficient strategy for newborn screening to reduce false-positive cases as well as to assess the efficacy of new therapeutic strategies.

## Figures and Tables

**Table 1:** Important molecular characteristics of galactosemia.

Forms of Galactosemia	Gene	Location	Gene Size	Exons	Variants	Comments
Type 1 galactosemia (OMIM 230400)	*GALT*	9p13.3	4.3 kb	11	Ser135Leu Lys285Asn Gln188Arg Asn314Asp del 5.5 kb del 5 kb	Common in African American populations [14] Prevalent in Southern Germany, Austria, and Croatia [15] Prevalent in Caucasian populations [15,16] Found on Duarte and Los Angeles galactosemia variants [17,18] Reported in patients of Ashkenazi Jewish ancestry [19,20]
Type 2 galactosemia (OMIM 230200)	*GALK1*	17q25.1	7.3 kb	8	Pro28Thr Ala198Val	Common in Gypsy populations [21] Common in Japanese and Korean populations [22]
Type 3 galactosemia (OMIM 230350)	*GALE*	1p36.11	5 kb	13	Val94Met Lys257Arg Gly319Glu	Reported in patients with generalized deficiency [23,24] Reported in African Americans with peripheral deficiency [23,24]
Type 4 galactosemia (OMIM 618881)	*GALM*	2p22.1	68 kb	7	Arg82* Ile99Leufs*46Trp311* Gly142Arg Arg267Gly	Reported in Japanese patients [25,26]

Data retrieved from ensembl.org, last accessed on 20 May 2022. The “*” is used to indicate a translational stop codon.

**Table 2:** Ongoing interventional clinical trials for novel treatments in galactosemias.

Condition	Drug	Approach	Participant Age	Study Phase	Study Identifier
CG	AT-007	Enzyme inhibitor	2–18	2/3	NCT04902781
CG	AT-007	Enzyme inhibitor	18–55	1/2	NCT04117711
CG	BBC	Behavioral	0.2–4.5	N.A.	NCT03838016
CG	N.A.	Cryopreservation	4–12	N.A.	NCT04948658

Data retrieved from Clinicaltrials.gov, last accessed on 23 May 2022. BBC: babble boot camp; CG: classic galactosemia; N.A.: not applicable.

## Data Availability

Not applicable.
